# Single-Domain Antibody Nuclear Imaging Allows Noninvasive Quantification of LAG-3 Expression by Tumor-Infiltrating Leukocytes and Predicts Response of Immune Checkpoint Blockade

**DOI:** 10.2967/jnumed.120.258871

**Published:** 2021-11

**Authors:** Quentin Lecocq, Robin Maximilian Awad, Yannick De Vlaeminck, Wout de Mey, Thomas Ertveldt, Cleo Goyvaerts, Geert Raes, Kris Thielemans, Marleen Keyaerts, Nick Devoogdt, Karine Breckpot

**Affiliations:** 1 Laboratory for Molecular and Cellular Therapy, Vrije Universiteit Brussel, Brussels, Belgium;; 2 Myeloid Cell Immunology Lab, VIB Center for Inflammation Research, Brussels, Belgium;; 3 Unit of Cellular and Molecular Immunology, Vrije Universiteit Brussel, Brussels, Belgium;; 4 In Vivo Cellular and Molecular Imaging Laboratory, Vrije Universiteit Brussel, Brussels, Belgium; and; 5 Nuclear Medicine Department, UZ Brussel, Brussels, Belgium

**Keywords:** cancer, nuclear imaging, single domain antibody, Nanobody, immune checkpoint, LAG-3

## Abstract

Recent advances in the field of immune-oncology led to the discovery of next-generation immune checkpoints (ICPs). Lymphocyte activation gene-3 (LAG-3), being the most widely studied among them, is being explored as a target for the treatment of cancer patients. Several antagonistic anti-LAG-3 antibodies are being developed and are prime candidates for clinical application. Furthermore, validated therapies targeting cytotoxic T-lymphocyte–associated protein-4, programmed cell-death protein-1, or programmed cell-death ligand-1 showed that only subsets of patients respond. This finding highlights the need for better tools for patient selection and monitoring. The potential of molecular imaging to detect ICPs noninvasively in cancer is supported by several preclinical and clinical studies. Here, we report on a single-domain antibody to evaluate whole-body LAG-3 expression in various syngeneic mouse cancer models using nuclear imaging. **Methods:** SPECT/CT scans of tumor-bearing mice were performed 1 h after injection with radiolabeled single-domain antibody. Organs and tumors of mice were isolated and evaluated for the presence of the radiolabeled tracer and LAG-3–expressing immune cells using a γ-counter and flow cytometry respectively. PD-1/LAG-3–blocking antibodies were injected in MC38-bearing mice. **Results:** The radiolabeled single-domain antibody detected LAG-3 expression on tumor-infiltrating lymphocytes (TILs) as soon as 1 h after injection in MC38, MO4, and TC-1 cancer models. The single-domain antibody tracer visualized a compensatory upregulation of LAG-3 on TILs in MC38 tumors of mice treated with PD-1–blocking antibodies. When PD-1 blockade was combined with LAG-3 blockade, a synergistic effect on tumor growth delay was observed. **Conclusion:** These findings consolidate LAG-3 as a next-generation ICP and support the use of single-domain antibodies as tools to noninvasively monitor the dynamic evolution of LAG-3 expression by TILs, which could be exploited to predict therapy outcome.

## Visual Abstract

**Figure:**
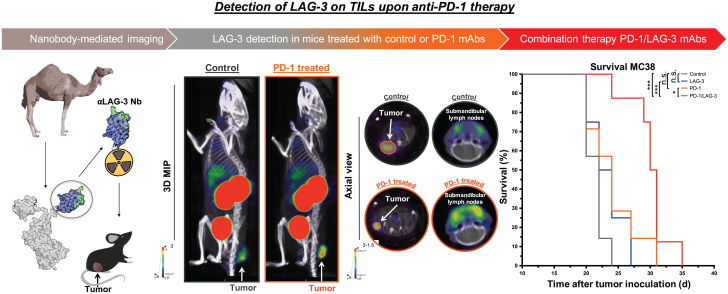

A frequently exploited immunotherapy strategy in cancer is blockade of inhibitory immune checkpoints (ICPs) (*1*). So far, the Food and Drug Administration has approved 7 antagonistic monoclonal antibodies (mAbs) against cytotoxic T-lymphocyte-associated protein-4 (CTLA-4), programmed cell-death protein-1 (PD-1), and its ligand PD-L1 for treatment of cancer (*2*). Although groundbreaking and effective in subsets of patients, the response to CTLA-4 and PD-1/PD-L1 mAbs is not satisfactory, as most patients show primary or acquired resistance (*3*). This observation instigated research into novel ICPs, which could compensate for the loss of the targeted ICP. Of these, lymphocyte activation gene-3 (LAG-3) is a promising target with a high probability of clinical success (*4*,*5*).

LAG-3 is a CD4-like molecule belonging to the immunoglobulin superfamily and is expressed on activated CD4^+^ and CD8^+^ T cells (*6*), regulatory T cells (*7*), B cells (*8*), natural killer cells (*9*), plasmacytoid dendritic cells (*10*), and myeloid cells such as macrophages (*11*). Molecules such as MHC-II (*12*), galectin-3 (*13*), LSECtin (*14*), α-synuclein (*15*), and fibrinogenlike protein-1 (*16*) can interact with LAG-3, with MHC-II being the canonic ligand. LAG-3 signaling is coopted in the tumor microenvironment (TME) to enable tumor cell escape.

Relatlimab was the first anti-LAG-3 mAb that entered clinical testing, as a monotherapy or combination therapy with nivolumab, an anti-PD-1 mAb, in melanoma, renal cell carcinoma, and non–small cell lung carcinoma (NCT019680109). This phase I trial showed that LAG-3 and PD-1 blockade is safe and restores T-cell functionality, leading to testing in phase II trials. Today, 7 other mAbs and 3 antibody derivatives that target LAG-3 are being evaluated in the clinic in a variety of malignancies. The high potential of LAG-3 blockade for cancer therapy is evidenced by the results in melanoma patients, who progressed despite previous immunotherapy not related to LAG-3 and who showed an 11% objective response rate when treated with relatlimab, with 1 complete and 6 partial responders (*17*). In this study, LAG-3 expression on nucleated cells within the tumor and invasive margin was determined using immunohistochemistry. It was observed that the objective response rate almost doubled in the LAG-3 expression group, underscoring the need for patient stratification.

Immunohistochemistry has several limitations with respect to ICP detection and subsequent patient selection (*18*,*19*). The need for a biopsy, the heterogeneous and dynamic expression of ICPs within the TME, and their role outside the TME are factors that can lead to incorrect stratification of patients. This could, for instance, explain why patients with undetectable PD-L1 levels showed beneficial effects of PD-L1 blockade (*20*). As opposed to immunohistochemistry, nuclear imaging is a noninvasive process that can be performed repeatedly irrespective of the tumor location (*18*,*19*,*21*). For example, imaging of PD-1 or PD-L1 using PET tracers has been performed in clinical trials, showing a better correlation with therapy outcome than immunohistochemistry (*22*,*23*). For the sake of safety and clinical practicality, nuclear imaging should be fast and generate high-contrast images, which can be achieved with small, stable, and soluble antigen-binding moieties.

Single-domain antibodies, the smallest antigen-binding fragment of camelid heavy-chain–only antibodies, are excellent tools to target proteins in the TME (*18*,*19*). Single-domain antibodies are small and easy to engineer and produce. Several radiolabeled single-domain antibodies are currently being evaluated in the clinic, including a ^99m^Tc-radiolabeled single-domain antibody that targets PD-L1, showing specific uptake in patients with non–small cell lung carcinoma at 2 h after injection (*24*–*27*). Therefore, single-domain antibodies targeting LAG-3 could be interesting diagnostic tools for noninvasive detection of LAG-3 before and after ICP treatment.

We previously reported the characterization of single-domain antibodies that target mouse LAG-3, showing that ^99m^Tc-labeled single-domain antibody 3132 is an excellent SPECT probe to specifically detect LAG-3 on immune cells and on tumor cells that were engineered to express high levels of LAG-3 (*28*). In this study, we assessed the ability of this single-domain antibody to image LAG-3 on tumor-infiltrating lymphocytes (TILs) in different mouse cancer models by SPECT/CT. We show that this single-domain antibody can accurately quantify LAG-3 levels in the TIL compartment. We moreover demonstrate the ability to detect LAG-3 upregulation on TILs in MC38 tumors of mice that were treated with anti-PD-1 mAbs. The enhanced therapy outcome in MC38-bearing mice treated with mAbs blocking PD-1 and LAG-3 corroborates the upregulation of LAG-3, as observed by nuclear imaging with the LAG-3 single-domain antibody.

## MATERIALS AND METHODS

### Mice, Cell Lines, and Reagents

Female, C57BL/6 mice (6–12 wk old) were purchased from Charles River. The institution’s ethical committee for use of laboratory animals approved the experiments. These were performed following the European guidelines for animal experimentation. MC38 mouse colorectal cancer cells and human embryonic kidney 293T cells were obtained from ATCC. MO4 melanoma cells were provided by Kenneth Rock (University of Massachusetts Medical School). These cells were cultured in Dulbecco modified Eagle’s medium supplemented with 10% fetal bovine serum (Tico Europe), 2 mM l-glutamine, and 100 U/mL penicillin with 100 µg/mL streptomycin. The TC-1 mouse lung cancer cells were provided by Tzyy-Choou Wu (Johns Hopkins University) and cultured in RPMI1640 medium, supplemented with 10% fetal clone I serum (Thermo Fisher Scientific), l-glutamine, 100 U/mL penicillin with 100 µg/mL streptomycin, 1 mM sodium pyruvate with nonessential amino acids, 12.5 mM D^+^-glucose, 5 mM 4-(2-hydroxyethyl)-1-piperazineethanesulfonic acid, and 50 µM β-mercaptoethanol. Culture media and supplements were from Sigma-Aldrich unless noted otherwise. A LAG-3–specific PerCP-eFluor710 or phycoerythrin-labeled antibody (clone eBioC9B7W; Biolegend) was used in flow cytometry. Antibodies from BD Biosciences were used to discriminate immune cells in flow cytometry: CD45.2-APC-Cy7 (clone 104), CD4-AF700 (clone RM4-5), CD8-V450 (clone 53-67), CD19-AF647 (clone 1D3), and F4/80-BB700 (clone T452342).

### Single-Domain Antibody Production and Quality Control

The LAG-3 single-domain antibody 3132 was selected from a panel of candidates for its ability to bind to mouse LAG-3 (*28*). Single-domain antibody R3B23, binding a multiple myeloma paraprotein (*29*), was used as a negative control. Single-domain antibody production and quality control were performed as previously described (*28*).

### Inoculation of Tumor Cells and Monitoring of Tumor Growth

Mice were subcutaneously injected with 3 × 10^5^ MC38, MO4, or TC-1 cells. Body weight, behavior, physical appearance, and tumor ulceration were examined daily. Tumor dimensions were measured every other day using a caliper to calculate the tumor volume: (length × width^2^)/2. All procedures were performed under isoflurane anesthesia (5% for induction and 2.5% for maintenance, with an oxygen flow of 1 L/min).

### ICP Blockade in Tumor-Bearing Mice

MC38-bearing mice were injected intraperitoneally with 10 mg/kg Ultra-LEAF mAbs (Biolegend) targeting mouse PD-1 (clone RPM1-14), mouse LAG-3 (clone C9B7W), an isotype-matched control (IC) for the PD-1 monotherapy (rat IgG2a, clone RTK2758), or a mixture of IC mAbs for the anti-PD-1/LAG-3 combination therapy (rat IgG1, clone RTK2071, and rat IgG2a, clone RTK2758).

### Single-Domain Antibody ^99m^Tc Labeling, SPECT/CT Imaging, Image Analysis, and Biodistribution Analysis

One hour before pinhole SPECT/small-animal CT imaging, the mice were intravenously injected with 5 µg of ^99m^Tc-labeled LAG-3 or control single-domain antibody, with, on average, 68.8 ± 6.8 MBq and 88.9 ± 5.1 MBq of injected activity, respectively. SPECT/CT imaging was performed using a VECTor SPECT/PET scanner (MILabs). SPECT imaging was performed with a 1.5-mm 75-pinhole general-purpose collimator, in spiral mode with 6 bed positions. The SPECT scan time for the total body was 15 min, 150 s per position. The CT scan time was 139 s in total, set to 60 kV and 615 mA. During all imaging procedures, the mice were anesthetized by intraperitoneal injection with 75 mg/kg ketamine hydrochloride and 1 mg/kg medetomidine (Ketamidor; Richter Pharma AG). Immediately after imaging, the organs of the killed mice were isolated and weighed. The submandibular lymph nodes were selected as representative lymph nodes. The organ-specific uptake of each radiotracer was measured using a Wizard^2^ γ-counter (PerkinElmer). The uptake in each organ was corrected for decay and calculated as the percentage of injected activity per gram (%IA/g). Image analysis was performed using AMIDE (Medical Image Data Examiner software) and HOROS medical imaging viewer (lesser general public license at https://horosproject.org/). Isolated organs were kept in MACS tissue storage solution (Miltenyi Biotec) when flow cytometry analysis was required.

### Single-Cell Preparation of Tumor and Spleen

Single-cell suspensions of tumors and spleens were prepared according to protocols 130-096-730 and 130-095-926 of Miltenyi Biotec. The tumors were cut into approximately 3-mm pieces and transferred to gentleMACS C tubes containing 5 mL of ice-cold RPMI1640 supplemented with 1,000 U/mL DNase I and 100 µL of collagenase I. Tumors and whole spleens were homogenized at 37°C for, respectively, 45 and 15 min using the gentleMACS Octo Dissociator (programs cus_37C_mImpTu2 and 37C_m_SDK_1, respectively). The cell suspension was filtered through a 70-µm filter (BD Falcon), washed with phosphate-buffered saline (Sigma-Aldrich), and centrifuged for 5 min at 1,500 rpm. Red blood cells were removed by resuspending the cell pellet with 5 mL of lysis buffer (0.16 M NH_4_Cl, 0.17 M Tris, pH 7.2). After 2 min of incubation, 10 mL of phosphate-buffered saline were added, and the cells were centrifuged, resuspended in cold phosphate-buffered saline, counted (CasyTon; Innovatis), and prepared for flow cytometry staining and analysis.

### Flow Cytometry

Staining cell-surface markers was previously described (*30*). Live/dead staining of the single-cell preparations was performed using ZombieAqua (BV510; Biolegend). Blockade of FcγII and FcγIII receptors (CD16/32 antibody, clone 93; BioLegend) and staining of surface makers was performed in phosphate-buffered saline containing 0.5% bovine serum albumin and 0.02% NaN_3_. The cells were acquired on a FACSCelesta or LSRFortessa flow cytometer (BD Biosciences). Data were analyzed using FlowJo X software (Tree Star).

### Statistics

Statistical analyses were performed with GraphPad Prism software (version 7.2). Data are represented as mean ± SD. *P *values were calculated using Mann–Whitney tests and Spearman correlation tests for flow cytometry and single-domain antibody biodistribution experiments or log rank tests for survival experiments. The asterisks in the figures indicate statistical significance as follows: *P < 0.05, **P < 0.01, ***P < 0.001, and ****P < 0.0001; n.s. indicates not significant.

## RESULTS

### Radiolabeled LAG-3 Single-Domain Antibodies Allow Imaging of LAG-3 in TME

**FIGURE 1.**
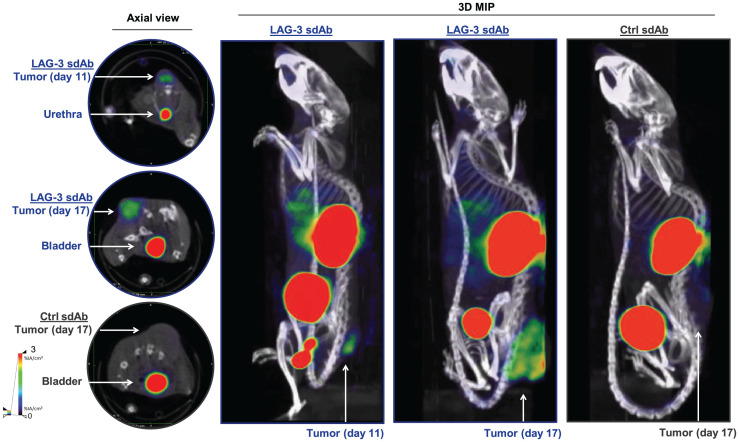
SPECT/CT imaging of LAG-3 using ^99m^Tc-labeled LAG-3 single-domain antibody in MC38-bearing mice at days 11 or 17 of tumor growth. Figure shows representative SPECT/CT images of MC38-bearing mice intravenously injected with ^99m^Tc-labeled LAG-3 or control single-domain antibodies. 3D MIP = 3-dimensional maximum-intensity projection; sdAb = single-domain antibody.

**FIGURE 2.**
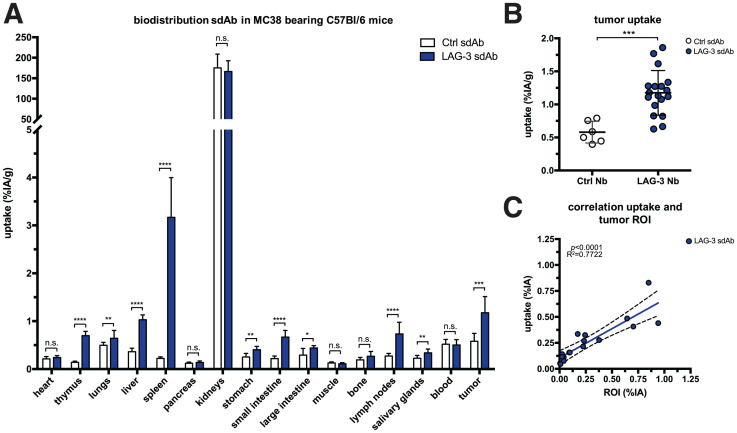
Biodistribution of radiolabeled LAG-3 and control single-domain antibodies in MC38-bearing mice at days 11 or 17 of tumor growth. (A) Ex vivo γ-counting of isolated organs from MC38-bearing mice 80 min after injection of LAG-3 or control single-domain antibody tracers. (B) Individual tumor uptake levels of LAG-3 and control single-domain antibody tracers as determined by ex vivo γ-counting. (C) Correlation plot of tumor uptake within image region of interest (*x*-axis) and ex vivo γ-counting of tumors (*y*-axis) for LAG-3 single-domain antibody tracer. ROI = region of interest; sdAb = single-domain antibody.

We radiolabeled LAG-3 or control single-domain antibodies with ^99m^Tc and compared their biodistribution in MC38-bearing mice by performing SPECT/CT at days 11 or 17 of tumor growth (Supplemental Figs. 1A–1C; supplemental materials are available at http://jnm.snmjournals.org). The average injected dose and tumor size at the time of evaluation are shown in Supplemental Figure 1D. Eighty minutes after injection, we observed signals in kidneys and bladder due to single-domain antibody clearance, and for the LAG-3 single-domain antibody tracer we observed signals in tumors (Fig. 1). After imaging, organs were dissected and weighed, and radioactivity levels were measured (Fig. 2A). Analysis of tumor uptake showed that only radiolabeled LAG-3 single-domain antibody accumulated in MC38 tumors, with little increase in larger tumors (*P* = 0.0364 and *P* = 0.0091 for day 11 and 17 tumors, respectively) (Fig. 2B). We assessed the accuracy of the radiolabeled single-domain antibodies to image LAG-3 expression levels in the tumor by comparing ex vivo γ-counts with the activity that can be measured on SPECT/CT scans using AMIDE software (Fig. 2C). LAG-3 single-domain antibody tumor uptake was also evaluated in mice bearing TC-1 or MO4 tumors. The average tumor size and the injected single-domain antibody doses are shown in Supplemental Figure 2A. Eighty minutes after injection, LAG-3 single-domain antibody accumulated in TC-1 and MO4 tumors (Supplemental Fig. 2B). Flow cytometry analysis showed LAG-3 expression on CD45^+^ immune cells in TC-1 or MO4 tumors (Supplemental Figs. 2C and 2D). In both models, tracer uptake levels and mean fluorescence intensity (MFI) of LAG3^+^ TILs correlated positively (Supplemental Fig. 2D). Representative axial images of both tumor types are shown in Supplemental Fig. 2E

### PD-1 Blockade Causes Upregulation of LAG-3 on TILs

We evaluated whether we could detect LAG-3 upregulation on TILs during PD-1 blockade in the MC38 model (*31*,*32*). Tumor-bearing mice were treated with anti-PD-1 or control mAbs. SPECT/CT imaging and ex vivo validations after injection of ^99m^Tc-labeled LAG-3 single-domain antibody were performed (Supplemental Fig. 3A).

**FIGURE 3.**
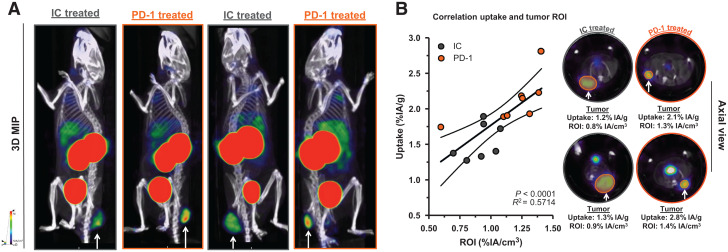
Effect of PD-1 blockade vs. IC in MC38-bearing mice on LAG-3 detection using ^99m^Tc-single-domain antibody SPECT/CT imaging. (A) Representative SPECT/CT images of mice treated with IC vs. PD-1–blocking mAbs. (B) Correlation plot and representative axial scans showing tumor signals in regions of interest (in %IA/cm^3^, *x*-axis) and ex vivo γ-counting of tumors from IC and PD-1–treated mice (in %IA/g, *y*-axis). 3D MIP = 3-dimensional maximum-intensity projection; ROI = region of interest.

**FIGURE 4.**
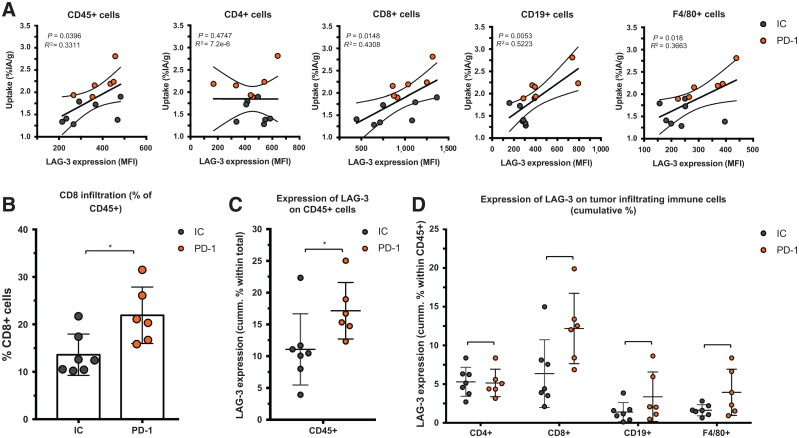
Ex vivo analysis of LAG-3 expression on immune cell populations within MC38 tumors of anti-PD-1–treated or IC-treated mice. (A) Correlation plot showing LAG-3 expression (MFI) on different immune cell subsets as analyzed by flow cytometry (*x*-axis) and ex vivo γ-counting results of tumors (%IA/g, *y*-axis). (B) Frequency of CD8^+^ T cells in MC38 tumors as analyzed by flow cytometry. (C and D) Expression of LAG-3 (cumulative %) on different immune cell subsets in MC38 tumors of anti-PD-1–treated (*n* = 6) or IC-treated (*n* = 7) mice as analyzed by flow cytometry.

**FIGURE 5.**
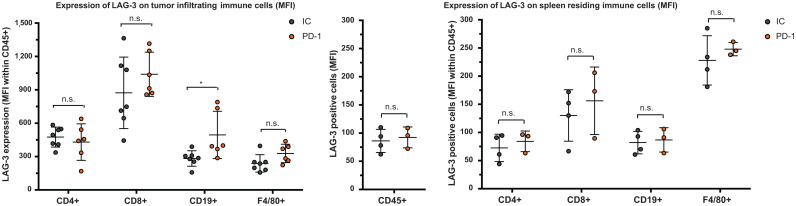
Ex vivo analysis of LAG-3 expression on immune cell populations within tumor or spleen of anti-PD-1–treated or IC-treated mice. Left graph illustrates expression of LAG-3 (MFI) on different immune cell subsets in MC38 tumors of anti-PD-1–treated (*n* = 6) or IC-treated (*n* = 7) mice, as analyzed by flow cytometry. Middle and right graphs illustrate expression of LAG-3 (MFI) on different immune cell subsets in spleens of anti-PD-1–treated (*n* = 3) or IC-treated (*n* = 4) mice, as analyzed by flow cytometry.

We observed a slower tumor growth when mice were treated with PD-1–blocking mAbs than when they were treated with IC mAbs (Supplemental Figs. 3B and 4A; *P = 0.0323*). We further analyzed SPECT/CT images using quantification software (Fig. 3A) and calculated activity within the region of interest, assigned over the tumor mass. These values correlated positively with the γ-counts obtained through ex vivo analysis (Fig. 3B). Ex vivo biodistribution analysis showed a significantly higher uptake of LAG-3 single-domain antibody in lymph nodes (*P* = 0.0037) and MC38 tumors (*P* = 0.0022), but not in spleen, as a result of PD-1 blockade (Supplemental Figs. 4B–4E, 5A, and 5B). LAG-3 single-domain antibody tumor uptake in mice treated with anti-PD-1 or IC mAbs was also compared with the MFI of LAG-3 expression on TILs, measured in flow cytometry. A positive correlation was found for CD45^+^ total TILs, CD8^+^ T cells, CD19^+^ B cells, and F4/80^+^ macrophages but not for CD4^+^ T cells (Fig. 4A). We also observed a significantly higher CD8^+^ T-cell infiltration in tumors of PD-1–treated mice relative to IC (Fig. 4B; *P* = 0.0350) but no differences in tumor infiltration for CD4^+^ T cells, CD19^+^ B cells, or F4/80^+^ macrophages (data not shown). Notably, we observed an increase in LAG-3 expression on total CD45^+^ TILs in mice treated with anti-PD-1 mAbs (*P* = 0.0221) (Fig. 4C). Although not significantly different between PD-1–treated and IC-treated groups, the proportion of LAG-3^+^ CD8^+^ T cells (*P* = 0.0518), CD19^+^ B cells (*P* = 0.1807), and F4/80^+^ macrophages (*P* = 0.1807) tended to be more pronounced in mice treated with anti-PD-1 mAbs (Fig. 4D). Elevated expression of LAG-3 on TILs in mice treated with anti-PD-1 mAbs was further corroborated by an increase in MFI, which was statistically different from the IC-treated group for CD19^+^ B cells (Fig. 5, *P* = 0.0140). Interestingly, no significant difference in LAG-3 expression was observed on spleen-residing immune cells of the PD-1–treated and IC-treated groups (Fig. 5), as is in line with the spleen uptake values of radiolabeled LAG-3 single-domain antibody (Supplemental Fig. 4D).

### Blockade of LAG-3 in Combination with PD-1 Blockade Enhances Therapy Outcome

**FIGURE 6.**
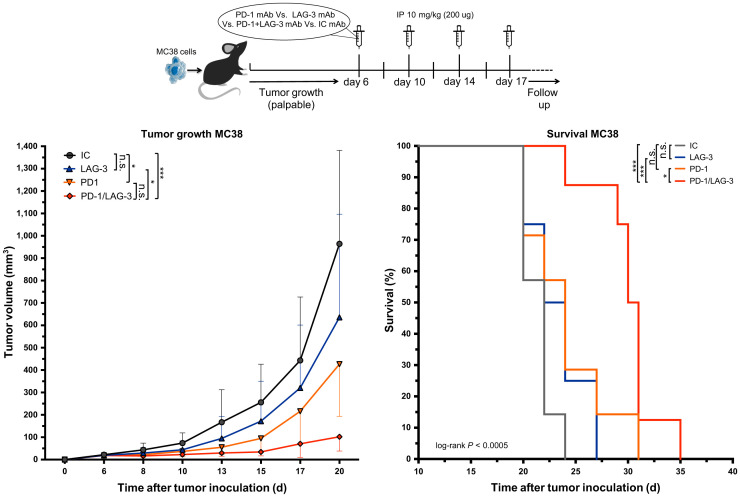
Response of established MC38 tumors to PD-1 or LAG-3 blockade. Top of figure illustrates timeline of experiment. Left curve displays growth kinetics of MC38 tumors treated with mAbs targeting PD-1 (*n* = 8), LAG-3 (*n* = 8), combination of PD-1 and LAG-3 (*n* = 8), or corresponding IC mAbs (*n* = 8). On right, Kaplan–Meyer survival curves are shown (time to reach humane endpoints, 1,500 mm^3^ tumor volume). IP = intraperitoneal.

We next assessed whether blockade of LAG-3 and PD-1 in the MC38 model prolonged the tumor growth delay. Tumor-bearing mice were treated with anti-PD-1 or anti-LAG-3 mAbs as shown in Figure 6. Mice treated with a mixture of IC mAbs served as a control. The delay in tumor growth on treatment with anti-LAG-3 mAbs alone was not significant, whereas a statistical difference was observed between anti-PD-1–treated and IC mAb–treated mice (Fig. 6; *P* = 0.0201). Combined PD-1/LAG-3 blockade further delayed tumor growth when compared with IC mAbs (*P* = 0.0001) or monotherapy with anti-LAG-3 (*P* = 0.0201) or anti-PD-1 (*P* = 0.5257) mAbs. This translated into a significantly longer time to reach humane endpoints in mice receiving the combination therapy than in mice treated with IC (*P* = 0.0005), anti-LAG-3 (*P* = 0.0004), or anti-PD-1 (*P* = 0.0181) mAbs (Fig. 6).

## DISCUSSION

mAbs targeting ICPs such as CTLA-4 and PD-1/PD-L1 have changed the field of immune-oncology because of their potent effects in a diversity of human cancers. However, there is still a fraction of patients who do not respond to this therapy. Additional ICPs such as LAG-3 have been discovered, offering the potential to overcome resistance in some of these patients by blocking LAG-3. Its important role in cancer development has been addressed in numerous preclinical studies (*28*,*32*–*37*). LAG-3 was shown to be expressed on T cells, B cells, plasmacytoid dendritic cells, natural killer cells, and macrophages (*1*,*6*,*11*,*28*). Its induction is related to the dysfunction of cancer-specific T cells often associated with PD-1 coexpression (*33*,*34*,*38*). The latter could be an explanation of therapeutic resistance to single-agent ICP blockade in patients. Subsequently, efforts have been made to develop anti-LAG-3 mAbs and explore their anticancer efficacy when used alone or in combination with anti-PD-1 blockade.

Evaluating the expression of ICPs within the tumor to select patients and predict their therapy outcome is of interest. Nuclear imaging using small binding moieties such as single-domain antibodies is a method to quickly detect biomarkers with high contrast in a noninvasive way (*18*,*21*). Moreover, this method can be performed repeatedly in the same patient regardless of the location of the tumor. Several clinical studies led to the observation that radiolabeled single-domain antibodies are good candidates to detect tumor biomarkers with high contrast as soon as 60 min after administration to the patient (*19*,*26*,*27*,*39*).

To our knowledge, we were the first to report different single-domain antibodies targeting mouse LAG-3 and to select one of these single-domain antibodies for detection of LAG-3–engineered tumor cells (*28*). In this study, we evaluated this single-domain antibody for detection of LAG-3–expressing TILs. We performed SPECT/CT imaging on immunocompetent colon carcinoma–bearing mice using ^99m^Tc-labeled LAG-3 single-domain antibody and compared tumor uptake values with a radiolabeled control single-domain antibody. Moreover, we performed imaging at days 11 or 17 of tumor growth to evaluate whether tumor size alters LAG-3 detection with the single-domain antibody tracer. Besides the specific signals detected in organs such as the thymus, spleen, and lymph nodes, we observed a significantly increased uptake of LAG-3 single-domain antibody in tumors compared with control single-domain antibody. No difference in single-domain antibody uptake was observed when comparing weight-corrected uptake values in the tumors imaged at days 11 or 17. The ex vivo measured tumor activities could be correlated to the SPECT/CT images. We further extended our SPECT/CT imaging studies and ex vivo validations to a syngeneic melanoma and lung cancer model. The LAG3 single-domain antibody tracer also accumulated in these tumors. Notably, uptake values correlated with the ex vivo–evaluated LAG-3 expression on CD45^+^ TILs using flow cytometry. Overall, the SPECT/CT images of these scans show the potency of the single-domain antibody tracer to map LAG-3 in the TME.

LAG-3 expression can act as a compensatory mechanism that leads to therapeutic resistance of PD-1 blockade in cancer patients (*17*,*33*,*34*,*38*). To our knowledge, we are the first to explore the effects of PD-1 treatment on LAG-3 expression and distribution using molecular imaging. MC38-bearing mice were treated with PD-1–blocking mAbs, leading to tumor growth impairment. Noninvasive imaging revealed elevated uptake of LAG-3 single-domain antibody tracer in tumor and lymph nodes relative to the IC treatment group. PD-1–treated mice had more CD8^+^ T cells in their tumor and more LAG-3^+^ TILs. A positive correlation was found between the amount of LAG-3 single-domain antibody tracer in the tumor and the LAG-3 levels on CD8^+^ T cells, CD19^+^ B cells, and F4/80^+^ macrophages. However, some individual samples were inconclusive when we attempted to correlate LAG-3 single-domain antibody uptake levels with flow cytometry data. Intriguingly, previous studies suggest that checkpoint blockade causes changes in the tumor environment such as a reduction in tumor interstitial pressure and decompressed tumor blood vessels (*40*). Although proven for PD-L1–blocking antibodies, we must not exclude the possibility of similar anti-PD-1–mediated modifications that can alter the exposure of tumors to our radiolabeled LAG-3 single-domain antibody tracer. Nevertheless, the proper correlation of LAG-3 single-domain antibody uptake levels with flow cytometry data could have been clarified by also analyzing tumors using immunohistochemistry, as it would have allowed us to more accurately quantify the percentage of immune cells per unit weight of tumor. Taken together, our findings support the idea of a compensatory upregulation of LAG-3 in the TME after PD-1 blockade. This compensatory LAG-3 expression in tumors on PD-1 treatment could be reliably captured with high contrast with the LAG-3 single-domain antibody SPECT/CT tracer.

Because we observed a compensatory upregulation of LAG-3 in the TME after single-agent PD-1 blockade, we evaluated the anticancer effects of combined LAG-3/PD-1 blockade in MC38 tumors. Tumor growth was marginally affected by LAG-3 blockade. However, when combined with PD-1 blockade, its anticancer effect surpasses the already significant decrease in tumor growth observed with PD-1 blockade. This corroborates the compensatory role of LAG-3 on PD-1 blockade in the MC38 model and underscores the benefit of combining blockade of multiple ICPs (*31*,*32*).

As an increasing number of clinical trials investigate LAG-3 blockade in immunotherapy, most often in combination with PD-1 blockade, a clinical tracer to monitor LAG-3 levels in the tumors of patients is also of high interest. This study used a single-domain antibody–binding mouse LAG-3 with no cross-reactivity with human LAG-3. We developed a single-domain antibody that binds to human LAG-3 with low-nanomolar affinity for human LAG-3 and a good capacity to visualize human LAG-3^+^ tumors in mice (Quentin Lecocq, unpublished data, August 2021). Optimally, this single-domain antibody needs to be labeled with a short-lived PET isotope such as ^68^Ga or ^18^F using new or established radiochemistry procedures (*25*,*41*–*43*). Extrapolating from our still-ongoing clinical trials with a **^68^**Ga-labeled anti–human epidermal growth factor receptor 2 single-domain antibody breast cancer PET tracer (*26*) and an anti-CD206 single-domain antibody macrophage PET tracer (*25*), we are hopeful that the future clinical anti-LAG-3 single-domain antibody PET tracer will also be safe and sensitive and will conveniently provide a whole-body picture of LAG-3 expression levels in a same-day imaging procedure with acceptable dosimetry levels.

## CONCLUSION

These findings consolidate LAG-3 as a next-generation ICP and support the use of single-domain antibodies as tools to noninvasively monitor the dynamic evolution of LAG-3 expression by TILs, which could be exploited to predict therapy outcome.

## DISCLOSURE

This research was financially supported by the Belgian Foundation against Cancer, Kom op tegen Kanker (Stand Up to Cancer), the Flemish Cancer Society, and the Research Foundation Flanders (FWO-V; 1501019N and I001618N). Quentin Lecocq has been funded via an “Emmanuel Vanderschueren” award and is an FWO-SB fellow (1S24218N). Marleen Keyaerts received research funding from Precirix (formerly named Camel-IDS). Quentin Lecocq, Karine Breckpot, Nick Devoogdt, and Marleen Keyaerts have patents on the use of single-domain antibodies for imaging and therapy. Nick Devoogdt and Marleen Keyaerts have ownership in AbScint, which leverages single-domain antibody imaging tracers into clinical application. No other potential conflict of interest relevant to this article was reported.

**KEY POINTS**

**QUESTION:** Is detection of LAG-3 using radiolabeled single-domain antibodies predictive of the therapeutic outcome of cancers treated with LAG-3–blocking moieties?
**PERTINENT FINDINGS:** This preclinical study describes the potential of a radiolabeled single-domain antibody to noninvasively detect the compensatory upregulation of LAG-3 as a consequence of PD-1 blockade in cancer. The combination of PD-1 with LAG-3 blockade was able to significantly reduce tumor growth compared with monotherapy.
**IMPLICATIONS FOR PATIENT CARE:** Single-domain antibodies, as tools to noninvasively monitor the dynamic evolution of LAG-3 expression by TILs, could be exploited to select patients and predict their therapy outcome.

## References

[1] Qin S Xu L Yi M Yu S Wu K Luo S. Novel immune checkpoint targets: moving beyond PD-1 and CTLA-4. Mol Cancer. 2019;18:155. 31690319 10.1186/s12943-019-1091-2 PMC6833286

[2] Li Z Song W Rubinstein M Liu D. Recent updates in cancer immunotherapy: a comprehensive review and perspective of the 2018 China Cancer Immunotherapy Workshop in Beijing. J Hematol Oncol. 2018;11:142. 30577797 10.1186/s13045-018-0684-3 PMC6303854

[3] O’Donnell JS Long GV Scolyer RA Teng MWL Smyth MJ. Resistance to PD1/PDL1 checkpoint inhibition. Cancer Treat Rev. 2017;52:71–81. 27951441 10.1016/j.ctrv.2016.11.007

[4] Andrews LP Marciscano AE Drake CG Vignali DAA. LAG3 (CD223) as a cancer immunotherapy target. Immunol Rev. 2017;276:80–96. 28258692 10.1111/imr.12519 PMC5338468

[5] Lecocq Q Keyaerts M Devoogdt N Breckpot K. The next-generation immune checkpoint LAG-3 and its therapeutic potential in oncology: third time’s a charm. Int J Mol Sci. 2020;22:75. 10.3390/ijms22010075 PMC7795594 33374804

[6] Triebel F Jitsukawa S Baixeras E. LAG-3, a novel lymphocyte activation gene closely related to CD4. J Exp Med. 1990;171:1393–1405. 1692078 10.1084/jem.171.5.1393 PMC2187904

[7] Huang C-T Workman CJ Flies D. Role of LAG-3 in regulatory T cells. Immunity. 2004;21:503–513. 15485628 10.1016/j.immuni.2004.08.010

[8] Kisielow M Kisielow J Capoferri-Sollami G Karjalainen K. Expression of lymphocyte activation gene 3 (LAG-3) on B cells is induced by T cells. Eur J Immunol. 2005;35:2081–2088. 15971272 10.1002/eji.200526090

[9] Huard B Tournier M Triebel F. LAG-3 does not define a specific mode of natural killing in human. Immunol Lett. 1998;61:109–112. 9657262 10.1016/s0165-2478(97)00170-3

[10] Andreae S Piras F Burdin N Triebel F. Maturation and activation of dendritic cells induced by lymphocyte activation gene-3 (CD223). J Immunol. 2002;168:3874–3880. 11937541 10.4049/jimmunol.168.8.3874

[11] Keane C Law SC Gould C. LAG3: a novel immune checkpoint expressed by multiple lymphocyte subsets in diffuse large B-cell lymphoma. Blood Adv. 2020;4:1367–1377. 32267932 10.1182/bloodadvances.2019001390 PMC7160288

[12] Baixeras E Huard B Miossec C. Characterization of the lymphocyte activation gene 3-encoded protein: a new ligand for human leukocyte antigen class II antigens. J Exp Med. 1992;176:327–337. 1380059 10.1084/jem.176.2.327 PMC2119326

[13] Kouo T Huang L Pucsek AB. Galectin-3 shapes antitumor immune responses by suppressing CD8+ T cells via LAG-3 and inhibiting expansion of plasmacytoid dendritic cells. Cancer Immunol Res. 2015;3:412–423. 25691328 10.1158/2326-6066.CIR-14-0150 PMC4390508

[14] Xu F Liu J Liu D. LSECtin expressed on melanoma cells promotes tumor progression by inhibiting antitumor T-cell responses. Cancer Res. 2014;74:3418–3428. 24769443 10.1158/0008-5472.CAN-13-2690

[15] Mao X Ou MT Karuppagounder SS. Pathological α-synuclein transmission initiated by binding lymphocyte-activation gene 3. Science. 2016;353:aah3374. 10.1126/science.aah3374 PMC5510615 27708076

[16] Wang J Sanmamed MF Datar I. Fibrinogen-like protein 1 is a major immune inhibitory ligand of LAG-3. Cell. 2019;176:334–347.e12. 30580966 10.1016/j.cell.2018.11.010 PMC6365968

[17] ESMO 2017 congress: 8-12 September 2017—Madrid, Spain. European Society for Medical Oncology website. https://oncologypro.esmo.org/content/download/126251/2385263/file/ESMO-2017-Congress-Scientific-Meeting-Report.pdf. Accessed April 28, 2021.

[18] Broos K Lecocq Q Raes G Devoogdt N Keyaerts M Breckpot K. Noninvasive imaging of the PD-1:PD-L1 immune checkpoint: embracing nuclear medicine for the benefit of personalized immunotherapy. Theranostics. 2018;8:3559–3570. 30026866 10.7150/thno.24762 PMC6037030

[19] Lecocq Q De Vlaeminck Y Hanssens H. Theranostics in immuno-oncology using nanobody derivatives. Theranostics. 2019;9:7772–7791. 31695800 10.7150/thno.34941 PMC6831473

[20] Teng MWL Ngiow SF Ribas A Smyth MJ. Classifying cancers based on T-cell infiltration and PD-L1. Cancer Res. 2015;75:2139–2145. 25977340 10.1158/0008-5472.CAN-15-0255 PMC4452411

[21] Du Y Jin Y Sun W Fang J Zheng J Tian J. Advances in molecular imaging of immune checkpoint targets in malignancies: current and future prospect. Eur Radiol. 2019;29:4294–4302. 30506221 10.1007/s00330-018-5814-3 PMC6610275

[22] Bensch F van der Veen EL Lub-de Hooge MN. ^89^Zr-atezolizumab imaging as a non-invasive approach to assess clinical response to PD-L1 blockade in cancer. Nat Med. 2018;24:1852–1858. 30478423 10.1038/s41591-018-0255-8

[23] Niemeijer AN Leung D Huisman MC. Whole body PD-1 and PD-L1 positron emission tomography in patients with non-small-cell lung cancer. Nat Commun. 2018;9:4664. 30405135 10.1038/s41467-018-07131-y PMC6220188

[24] Broisat A Toczek J Dumas LS. ^99m^Tc-cAbVCAM1-5 imaging is a sensitive and reproducible tool for the detection of inflamed atherosclerotic lesions in mice. J Nucl Med. 2014;55:1678–1684. 25157043 10.2967/jnumed.114.143792

[25] Xavier C Blykers A Laoui D. Clinical translation of [^68^Ga]Ga-NOTA-anti-MMR-sdAb for PET/CT imaging of protumorigenic macrophages. *Mol Imaging Biol* . 2019;21:898–906. 10.1007/s11307-018-01302-5 30671739

[26] Keyaerts M Xavier C Heemskerk J. Phase I study of ^68^Ga-HER2-nanobody for PET/CT assessment of HER2 expression in breast carcinoma. J Nucl Med. 2016;57:27–33. 26449837 10.2967/jnumed.115.162024

[27] Xing Y Chand G Liu C. Early phase I study of a ^99m^Tc-labeled anti-programmed death ligand-1 (PD-L1) single-domain antibody in SPECT/CT assessment of PD-L1 expression in non-small cell lung cancer. J Nucl Med. 2019;60:1213–1220. 30796165 10.2967/jnumed.118.224170 PMC6735283

[28] Lecocq Q Zeven K De Vlaeminck Y. Noninvasive imaging of the immune checkpoint LAG-3 using nanobodies, from development to pre-clinical use. Biomolecules. 2019;9:548. 10.3390/biom9100548 PMC6843898 31569553

[29] Lemaire M D’Huyvetter M Lahoutte T. Imaging and radioimmunotherapy of multiple myeloma with anti-idiotypic Nanobodies. Leukemia. 2014;28:444–447. 24166214 10.1038/leu.2013.292

[30] Breckpot K Dullaers M Bonehill A. Lentivirally transduced dendritic cells as a tool for cancer immunotherapy. J Gene Med. 2003;5:654–667. 12898635 10.1002/jgm.400

[31] Yu X Huang X Chen X. Characterization of a novel anti-human lymphocyte activation gene 3 (LAG-3) antibody for cancer immunotherapy. MAbs. 2019;11:1139–1148. 10.1080/19420862.2019.1629239 PMC6748621 31242068

[32] Burova E Hermann A Dai J. Preclinical development of the anti-LAG-3 antibody REGN3767: characterization and activity in combination with the anti-PD-1 antibody cemiplimab in human PD-1xLAG-3-knockin mice. Mol Cancer Ther. 2019;18:2051–2062. 31395688 10.1158/1535-7163.MCT-18-1376

[33] Huang R-Y Francois A McGray AR Miliotto A Odunsi K. Compensatory upregulation of PD-1, LAG-3, and CTLA-4 limits the efficacy of single-agent checkpoint blockade in metastatic ovarian cancer. OncoImmunology. 2016;6:e1249561. 28197366 10.1080/2162402X.2016.1249561 PMC5283642

[34] Huang R-Y Eppolito C Lele S Shrikant P Matsuzaki J Odunsi K. LAG3 and PD1 co-inhibitory molecules collaborate to limit CD8+ T cell signaling and dampen antitumor immunity in a murine ovarian cancer model. Oncotarget. 2015;6:27359–27377. 26318293 10.18632/oncotarget.4751 PMC4694995

[35] Woo S-R Turnis ME Goldberg MV. Immune inhibitory molecules LAG-3 and PD-1 synergistically regulate T-cell function to promote tumoral immune escape. Cancer Res. 2012;72:917–927. 22186141 10.1158/0008-5472.CAN-11-1620 PMC3288154

[36] Harris-Bookman S Mathios D Martin AM. Expression of LAG-3 and efficacy of combination treatment with anti-LAG-3 and anti-PD-1 monoclonal antibodies in glioblastoma. Int J Cancer. 2018;143:3201–3208. 30248181 10.1002/ijc.31661 PMC7105259

[37] Matsuzaki J Gnjatic S Mhawech-Fauceglia P. Tumor-infiltrating NY-ESO-1-specific CD8+ T cells are negatively regulated by LAG-3 and PD-1 in human ovarian cancer. Proc Natl Acad Sci USA. 2010;107:7875–7880. 20385810 10.1073/pnas.1003345107 PMC2867907

[38] Nagasaki J Togashi Y Sugawara T. The critical role of CD4+ T cells in PD-1 blockade against MHC-II-expressing tumors such as classic Hodgkin lymphoma. Blood Adv. 2020;4:4069–4082. 32870971 10.1182/bloodadvances.2020002098 PMC7479950

[39] Rashidian M Ploegh H. Nanobodies as non-invasive imaging tools. Immuno-Oncology Technol. 2020;7:2–14. 10.1016/j.iotech.2020.07.001 PMC9216400 35754459

[40] Clift R Souratha J Garrovillo SA Zimmerman S Blouw B. Remodeling the tumor microenvironment sensitizes breast tumors to anti-programmed death-ligand 1 immunotherapy. Cancer Res. 2019;79:4149–4159. 31248966 10.1158/0008-5472.CAN-18-3060

[41] Bridoux J Broos K Lecocq Q. Anti-human PD-L1 Nanobody for immuno-PET imaging: validation of a conjugation strategy for clinical translation. Biomolecules. 2020;10:1388. 10.3390/biom10101388 PMC7599876 33003481

[42] Cleeren F Lecina J Ahamed M. Al^18^F-labeling of heat-sensitive biomolecules for positron emission tomography imaging. Theranostics. 2017;7:2924–2939. 28824726 10.7150/thno.20094 PMC5562226

[43] Blykers A Schoonooghe S Xavier C. PET imaging of macrophage mannose receptor-expressing macrophages in tumor stroma using ^18^F-radiolabeled camelid single-domain antibody fragments. J Nucl Med. 2015;56:1265–1271. 26069306 10.2967/jnumed.115.156828

